# Emergency management of ceftriaxone-induced Type II Kounis syndrome: A case report

**DOI:** 10.1097/MD.0000000000042917

**Published:** 2025-06-20

**Authors:** Jiali Wang, Zhenhua Jiang

**Affiliations:** a Shaoxing Central Hospital, Shaoxing, Zhejiang Province, China.

**Keywords:** acute coronary syndrome, anaphylactic shock, ceftriaxone, Kounis syndrome

## Abstract

**Rationale:**

Ceftriaxone, a broad-spectrum β-lactam antibiotic, can induce severe allergic reactions such as anaphylaxis, which may lead to life-threatening type II Kounis syndrome (K-S), a variant of acute coronary syndrome triggered by allergic reactions. This case report aims to enhance the clinical understanding of ceftriaxone-induced type II K-S by presenting a detailed account of a patient’s emergency management and subsequent recovery, emphasizing the importance of early recognition, intervention, and the role of nursing care in achieving a favorable outcome.

**Patient concerns:**

A 73-year-old male with no known allergy history was admitted for cough and shortness of breath. After ceftriaxone administration, he developed chest tightness, shortness of breath, hypotension, and abdominal rash.

**Diagnoses:**

Type II K-S triggered by ceftriaxone-induced anaphylaxis was diagnosed.

**Interventions:**

Immediate management included discontinuing ceftriaxone, emergency tracheal intubation, and intravenous epinephrine. Emergency coronary angiography showed no significant coronary artery stenosis. Postoperatively, the patient received antiplatelet therapy, plaque stabilization, and vasodilation in the intensive care unit.

**Outcomes:**

The patient’s condition stabilized, tracheal intubation was removed, and he was discharged after 10 days of treatment with significant clinical improvement.

**Lessons:**

This case emphasizes the critical importance of early recognition and management of ceftriaxone-induced type II K-S. Vigilant monitoring for allergic reactions and a multidisciplinary approach to care are essential for achieving favorable patient outcomes.

## 1. Introduction

Ceftriaxone, a third-generation cephalosporin, is widely recognized for its broad-spectrum antibacterial capabilities, potent efficacy, and extended half-life, positioning it as a cornerstone in clinical antibiotic therapy. Despite its widespread use and therapeutic advantages, ceftriaxone has been implicated in a spectrum of adverse reactions, with allergic responses being of particular concern.^[1]^ Notably, anaphylactic reactions to ceftriaxone are disproportionately prevalent compared to other antibiotics, as evidenced by pharmacovigilance data, and can lead to severe and life-threatening complications, including Type II Kounis syndrome (K-S).^[2,3]^

K-S, first delineated by Kounis and Zavras in 1991, is a clinical condition characterized by the concurrence of acute coronary syndrome with an allergic reaction.^[4]^ This syndrome can be triggered by a variety of stimuli, such as medications, foods, and environmental factors. K-S is classified into 3 subtypes: Type I, affecting those with normal coronary arteries and presenting with allergic angina; Type II, impacting individuals with underlying coronary disease, leading to allergic myocardial infarction; and Type III, specific to post-stent patients experiencing thrombosis due to allergic reactions.^[5,6]^

The incidence of ceftriaxone-induced allergic reactions, particularly anaphylaxis, is significant, and when such reactions occur in conjunction with ceftriaxone administration, they can precipitate K-S, leading to considerable morbidity and mortality. Management of this syndrome is complex and necessitates a multidisciplinary approach, including immediate discontinuation of the causative agent, aggressive anti-allergic treatment, and prompt cardiovascular support.^[1]^

Given the propensity of ceftriaxone to elicit severe allergic reactions and the critical implications of Type II K-S, it is imperative for healthcare providers to be vigilant for signs and symptoms of allergic responses and to be equipped to manage the concomitant cardiovascular complications effectively. This case report aims to enhance the clinical understanding of ceftriaxone-induced Type II K-S by presenting a detailed account of a patient’s emergency management and subsequent recovery, emphasizing the importance of early recognition, intervention, and the role of nursing care in achieving a favorable outcome. A brief literature review reveals that while ceftriaxone is a valuable asset in antimicrobial therapy, its potential to trigger severe allergic reactions necessitates a keen awareness and readiness to manage the associated cardiovascular emergencies.

## 2. Case presentation

**Figure 1.**
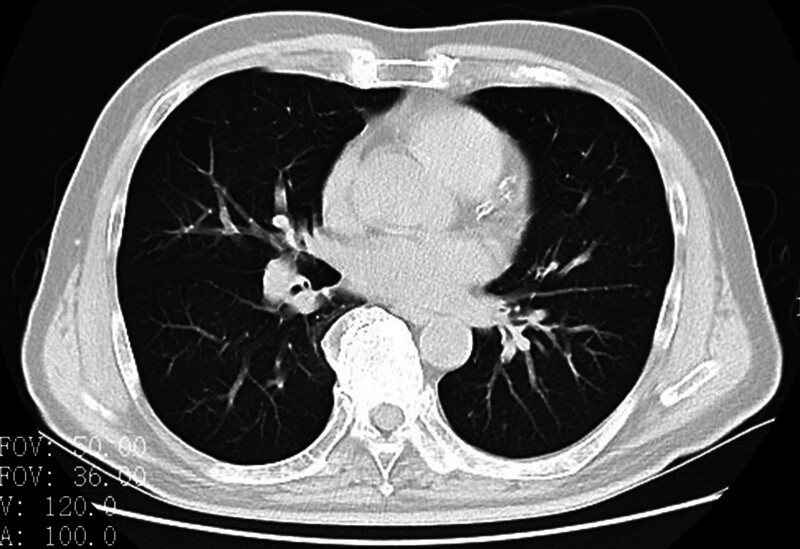
Computed tomography of the chest.

**Figure 2.**
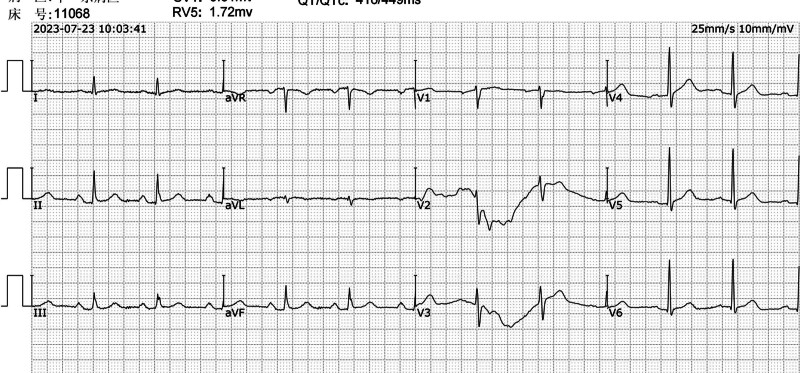
Electrocardiogram upon admission.

A 73-year-old male with no known allergy history was admitted to our hospital on July 23, 2023, for a 5-day history of cough and shortness of breath. Upon admission, his vital signs were stable. Physical examination revealed bilateral wheezing and mild pitting edema in both lower limbs. Initial diagnostic workup, including chest computed tomography and electrocardiogram (ECG), demonstrated minor fibrotic foci in both lungs, pleural changes, and calcified plaques in the coronary and aortic walls (Fig. 1). The ECG showed sinus rhythm with a first-degree atrioventricular block (Fig. 2).

## 3. Timeline of events

July 23, 2023 (admission): The patient presented with cough and shortness of breath. Initial assessments and diagnostic tests were performed.

Ceftriaxone administration: The patient received ceftriaxone for a suspected bacterial infection.

**Figure 3.**
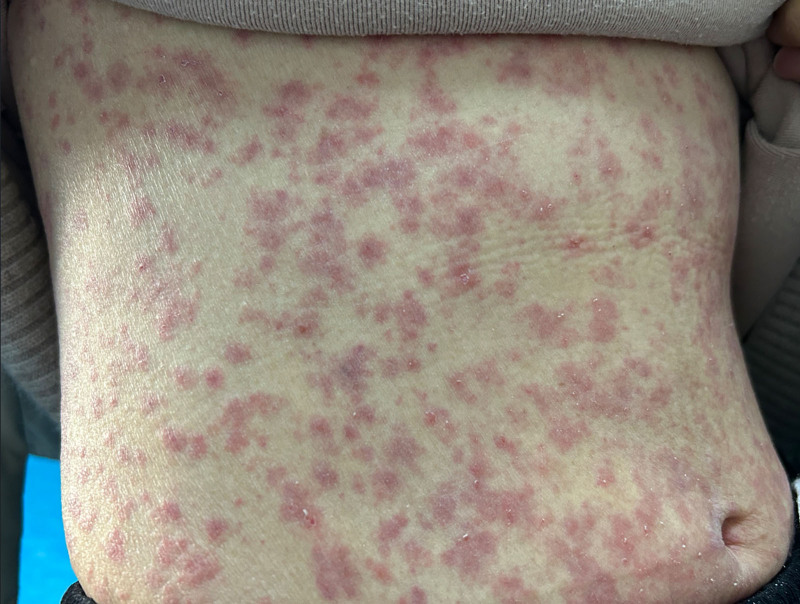
Abdominal rash.

**Figure 4.**
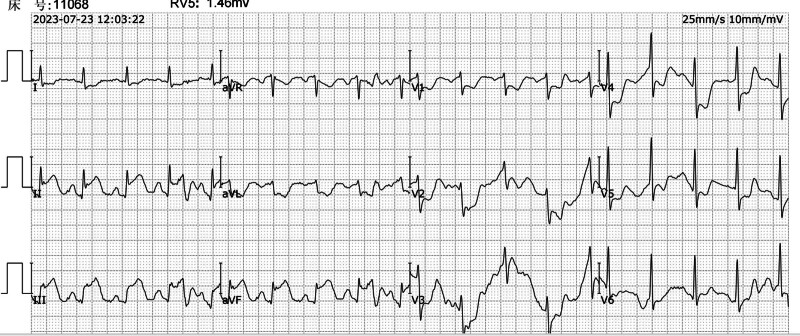
Electrocardiogram: suggestive of acute inferior myocardial infarction.

30 minutes post-ceftriaxone: The patient developed chest tightness, shortness of breath, hypotension, and an abdominal rash (Fig. 3). An ECG at this time revealed elevated ST segments in the inferior leads, indicative of myocardial ischemia (Fig. 4). Additionally, troponin I levels were measured at 0.1 ng/mL, further supporting the diagnosis of myocardial injury.

Immediate intervention: Given the respiratory distress, emergency tracheal intubation was performed. For the anaphylactic shock, subcutaneous epinephrine (0.3 mg) was administered, followed by fluid resuscitation and inotropic support with dopamine (3 mg intravenously) and norepinephrine (0.2 μg/[kg·min]) to maintain blood pressure. The patient was transported to the catheterization laboratory for emergency coronary angiography.

**Figure 5.**
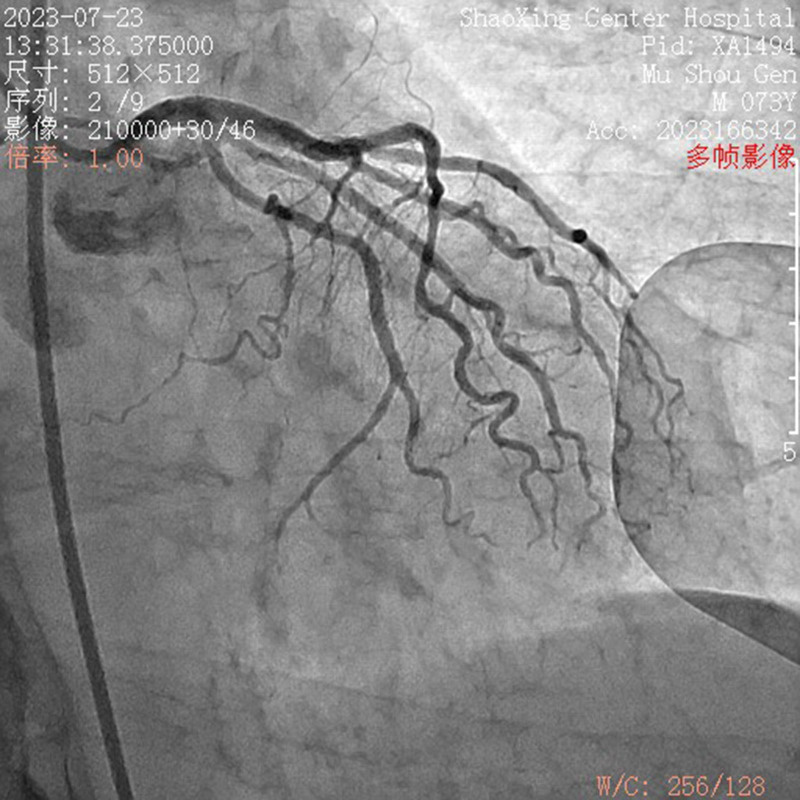
Left coronary arteriography.

**Figure 6.**
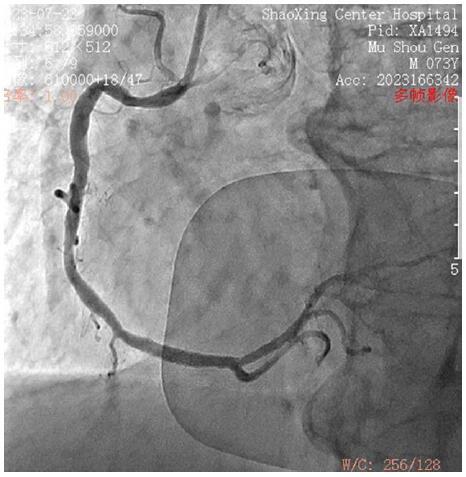
Right coronary arteriography.

Coronary angiography: The angiography showed no significant coronary artery stenosis but revealed a plaque in the middle segment of the anterior descending artery and stenosis in the right coronary artery (50% in the proximal segment and 60% in the middle segment) (Figs. 5 and 6).

Post-angiography management: The patient was transferred to the intensive care unit (ICU) for further treatment, which included antiplatelet therapy (aspirin 100 mg daily and clopidogrel 75 mg daily), plaque stabilization (atorvastatin 20 mg nightly), and vasodilation (nitroglycerin 0.3 mg sublingually as needed).

**Figure 7.**
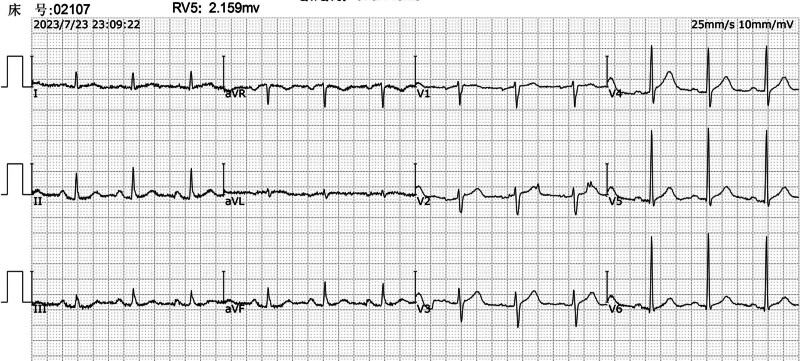
Postoperative electrocardiogram.

ICU stay: The patient’s condition stabilized, and the tracheal intubation was removed once his respiratory function improved. A follow-up ECG showed normalization of the ST segments (Fig. 7).

Discharge: After 10 days of comprehensive treatment, the patient exhibited significant clinical improvement without further complications and was subsequently discharged.

## 4. Key diagnostic findings

1. ECG changes: The initial ECG indicated sinus rhythm with a first-degree atrioventricular block (Fig. 2). During the allergic reaction, the ECG demonstrated elevated ST segments in the inferior leads, consistent with acute myocardial ischemia (Fig. 4). Posttreatment, the ECG showed resolution of the ST-segment elevation (Fig. 7).
2. Laboratory findings: Elevated troponin levels confirmed myocardial injury, aligning with the diagnosis of Type II K-S.
3. Rash: The presence of an abdominal rash supported the diagnosis of an allergic reaction (Fig. 3).

## 5. Discussion

The present case of a 73-year-old male with no known allergy history who developed Type II K-S following ceftriaxone administration highlights several critical aspects of drug-induced allergic reactions and their cardiovascular complications. This discussion aims to contextualize our case within the existing literature, emphasizing its uniqueness and clinical significance.

Firstly, the prompt recognition of anaphylactic symptoms and the subsequent diagnosis of K-S are crucial. The initial symptoms of chest tightness and dyspnea, along with elevated troponin I levels (0.1 ng/mL), suggested a cardiac event. However, the absence of significant stenosis in the coronary arteries during emergency angiography confirmed the diagnosis of K-S. This aligns with the latest guidelines on the diagnosis and management of K-S and acute coronary syndromes. The European Society of Cardiology and the American Heart Association emphasize the importance of considering alternative diagnoses when coronary angiography does not reveal significant stenosis in patients presenting with symptoms suggestive of acute coronary syndrome.^[7,8]^

Secondly, the management of our patient was prompt and multifaceted, involving the immediate cessation of the causative antibiotic, aggressive anti-allergic treatment with subcutaneous epinephrine (0.3 mg), fluid resuscitation, and inotropic support with dopamine (3 mg intravenously) and norepinephrine (0.2 μg/[kg·min]). This approach is in line with current recommendations for managing anaphylaxis and anaphylactic shock. The emergency tracheal intubation was a critical intervention due to the patient’s respiratory distress, underscoring the necessity for rapid airway management in such cases.^[9,10]^

Thirdly, this case illustrates the complexity of managing K-S even in the absence of significant coronary artery stenosis. The use of emergency coronary angiography served to rule out coronary artery spasm or thrombosis as the cause of the patient’s symptoms, which is consistent with the recommended approach when K-S is suspected.^[11–13]^ The procedure revealed no significant stenosis, thus confirming the diagnosis of K-S and aligning with the latest guidelines on the assessment and management of acute coronary syndromes.

When compared to previously reported cases of ceftriaxone-induced K-S, our case has unique features. S Ali reported a case where K-S developed 3 hours after ceftriaxone administration in a patient without dermatological symptoms, leading to diagnostic challenges, and symptoms improved with dexamethasone.^[14]^ Our patient rapidly developed severe symptoms within 30 minutes of ceftriaxone administration. Coronary angiography showed no significant stenosis, suggesting a Type 1 hypersensitivity reaction. The patient’s condition was critical but improved after subcutaneous adrenaline and vasoactive drugs. Notably, plaque was found in the right coronary artery, and it is possible that the allergen activated macrophages within the plaque, triggering an allergic reaction. Inflammatory mediators were released, causing vasodilation, hypotension, and impacting coronary artery flow, while also inducing coronary artery spasm.

The clinical implications of this case are profound. It emphasizes the need for healthcare providers to maintain a high index of suspicion for K-S in patients presenting with acute coronary syndrome symptoms, particularly following exposure to known allergens such as ceftriaxone. The timely discontinuation of the causative agent and the initiation of appropriate treatment are critical to preventing adverse outcomes. Additionally, the role of emergency coronary angiography in confirming the diagnosis and guiding further management cannot be overstated.

## 6. Conclusions

In conclusion, this case report reinforces the importance of a high index of suspicion for ceftriaxone-induced allergic reactions and the potential for K-S in patients presenting with chest pain and respiratory symptoms. It also underscores the need for a comprehensive, multidisciplinary approach to management, including the timely use of coronary angiography for diagnosis and the application of evidence-based treatment protocols for anaphylaxis and acute coronary syndromes.

## 7. Patient perspective

As a 73-year-old man, I never expected that a routine hospital visit for a persistent cough and shortness of breath would turn into such a life-threatening experience. I had been feeling unwell for about 5 days, with a cough that produced yellow sputum and some mild swelling in my legs. I decided to seek medical help, thinking it might be a simple infection that could be treated with antibiotics.

When I arrived at the hospital, the doctors ran some tests, including a chest computed tomography and an ECG. They told me there were some minor issues in my lungs and some calcified plaques in my coronary arteries, but nothing too serious. I was given ceftriaxone, an antibiotic, to treat what they suspected was a bacterial infection. However, just 30 minutes after receiving the medication, I started feeling extremely unwell. My chest tightened, I struggled to breathe, and I felt like my blood pressure dropped suddenly. I also noticed a rash developing on my abdomen. I was terrified (I had never experienced anything like this before).

The medical team acted quickly. They stopped the ceftriaxone immediately and rushed me into emergency care. I remember being intubated to help me breathe, and I was given epinephrine to stabilize my blood pressure. The doctors suspected that I was having a severe allergic reaction, which had triggered something called Type II K-S, a condition where an allergic reaction causes heart problems. I was taken for an emergency coronary angiography, which thankfully showed no severe blockages in my heart, but confirmed the diagnosis of K-S.

The next few days were a blur. I was in the ICU, receiving treatment to stabilize my heart and manage the allergic reaction. The doctors and nurses were incredibly attentive, monitoring me closely and adjusting my medications as needed. Slowly, I started to feel better. My breathing improved, and the rash disappeared. After 10 days in the hospital, I was finally discharged, feeling much stronger and grateful for the care I received.

Looking back, this experience was a wake-up call. I had no history of allergies, and I never imagined that a common antibiotic could cause such a severe reaction. I’m now much more aware of the importance of monitoring for allergic reactions, especially when starting new medications. I’m also deeply thankful for the quick and skilled response of the medical team, who saved my life. This experience has taught me to be more vigilant about my health and to always communicate any unusual symptoms to my doctors.

In the end, I’m just glad to be alive and recovering. I hope my story can help raise awareness about the potential risks of allergic reactions to medications like ceftriaxone and the importance of prompt medical intervention in cases like mine.

## Author contributions

**Conceptualization:** Zhenhua Jiang.

**Data curation:** Zhenhua Jiang.

**Formal analysis:** Jiali Wang.

**Investigation:** Jiali Wang.

**Software:** Jiali Wang.

**Writing – original draft:** Jiali Wang.
